# Congestive Heart Failure: Experimental Model

**DOI:** 10.3389/fped.2013.00033

**Published:** 2013-10-28

**Authors:** Antonio Francesco Corno, Xue Cai, Caroline B. Jones, Giuseppina Mondani, Mark R. Boyett, Jonathan Charles Jarvis, George Hart

**Affiliations:** ^1^School of Medical Sciences, Health Campus, University Sains Malaysia, Kubang Kerian, Kelantan, Malaysia; ^2^Core Technology Facility, University of Manchester, Manchester, UK; ^3^Alder Hey Children NHS Foundation Trust, Liverpool, UK; ^4^John Moores University, Liverpool, UK

**Keywords:** congestive heart failure, experimental research, left heart failure, ventricular dilatation, ventricular hyprtrophy

## Abstract

**Introduction:** Surgically induced, combined volume and pressure overload has been used in rabbits to create a simplified and reproducible model of acute left ventricular (LV) failure.

**Materials and Methods:** New Zealand white male rabbits (*n* = 24, mean weight 3.1 ± 0.2 kg) were randomly assigned to either the Control group (*n* = 10) or to the Heart Failure group (HF, *n* = 14). Animals in the Control group underwent “sham” procedures. Animals in the HF group underwent procedures to induce LV volume overload by inducing severe aortic valve regurgitation with aortic cusp disruption and pressure overload using an occlusive silver clip positioned around the pre-renal abdominal aorta.

**Results:** Following *Procedure-1* (volume overload) echocardiography confirmed severe aortic regurgitation in all animals in the HF group, with increased mean pulse pressure difference from 18 ± 3 to 38 ± 3 mmHg (*P* < 0.0001). After *Procedure-2* (pressure overload) all animals in the HF group showed clinical and echocardiographic signs of constriction of the abdominal aorta and echocardiography confirmed progressively declining LV function. At the end of the protocol there was a significant increase of the heart/body weight ratio in the HF group vs. Control group (4.6 ± 0.2 vs. 2.9 ± 0.1 g/kg, *P* < 0.05), and echocardiography showed in HF group significant increase of the LV end-diastolic diameter (2.15 ± 0.09 vs. 1.49 ± 0.03 cm, *P* < 0.001) and reduction of the LV shortening fraction (26.3 ± 3.8 vs. 41.3 ± 1.6%, *P* < 0.001).

**Conclusion:** This experimental model: (a) consistently produces LV hypertrophy/dilatation and subsequent congestive heart failure, (b) provides new data on the time course of LV dilatation, hypertrophy and failure, (c) allows study of the progress and evolution of LV systolic and diastolic dysfunction in the presence of induced LV failure, (d) is suitable to study intervention or pharmacological administration to reduce the negative effects of acute LV failure.

## Introduction

Left ventricular (LV) dilatation and hypertrophy have been created in experimental animal models by inducing LV volume or pressure overload.

In the last two decades the induction of experimental aortic valve regurgitation has been used to induce LV volume overload (1–3).

Several studies have been reported with this experimental model of volume overload induced by aortic valve regurgitation to evaluate systolic and diastolic ventricular function, the development of myocardial fibrosis and myocyte degeneration, and the effects of various medications (4–9).

Left ventricular hypertrophy has been studied in other experimental studies on animals after inducing systemic reno-vascular hypertension by clipping one of the renal arteries (10, 11).

This experimental model of renal hypertension with renal artery clipping has been used to study variations of biochemical and humoral factors as well as changes in the aortic wall and in the myocardium (12–20).

As an alternative method to induce LV hypertrophy, induction of proximal systemic hypertension has been obtained by narrowing the abdominal aorta with various technical procedures (21–26).

This experimental model of proximal systemic hypertension and subsequent LV hypertrophy has allowed studies on arterial wall reactivity and remodeling, endothelial nitric oxide production, renal function, adrenergic response, myocardial oxidative stress, and macrophage migration (21–33).

The combination of experimental volume and pressure overload of the left ventricle has been less frequently used in previous animal models, predominantly to study electrophysiology, and rhythm disturbances (34–37).

We report the use of a model of surgically induced, combined volume, and pressure overload in rabbits.

Previous authors described a 2-stage model of heart failure in the rabbit, with sacrifice of the animals at 6 weeks after the first operation (34). We have introduced changes in the protocol extending the observation period to 8 weeks after the operation and characterizing the model using in particular echocardiography and micro-CT scanning.

We present our simplified and reproducible model, suitable for any of the following studies:

(a) myocardial and vascular structure and ultra-structure, metabolism and function, during and after the development of left heart failure;
(b) effects of the combined volume and pressure overload on the conduction tissues and potential development of arrhythmias;
(c) potential prevention of myocardial and vascular remodeling due to combined volume and pressure overload;
(d) treatment of left heart failure with any type and/or combination of interventions and/or medications.

## Materials and Methods

### Experimental animal model

**Figure 1:**
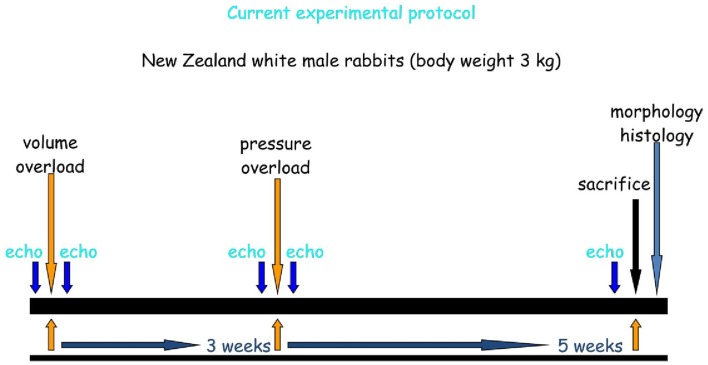
**Experimental protocol**.

New Zealand white male rabbits (*n* = 24) were arbitrarily assigned to either the Control group (*n* = 10) or to the HF group (*n* = 14). Animals in the Control group underwent “sham” procedures, while animals in the HF group underwent the procedures to induce LV volume overload and pressure overload. The entire protocol is summarized in Figure 1.

### Procedure-1 = volume overload

All rabbits were anesthetized with ketamine (50 mg/kg) and maintained with 2% isofluorane, after tracheal intubation and with self-ventilation.

Color Doppler echocardiography was performed with a GE Vivid 3 ultrasound recorder using a 5-MHz transducer in all animals before the surgical procedure.

**Figure 2:**
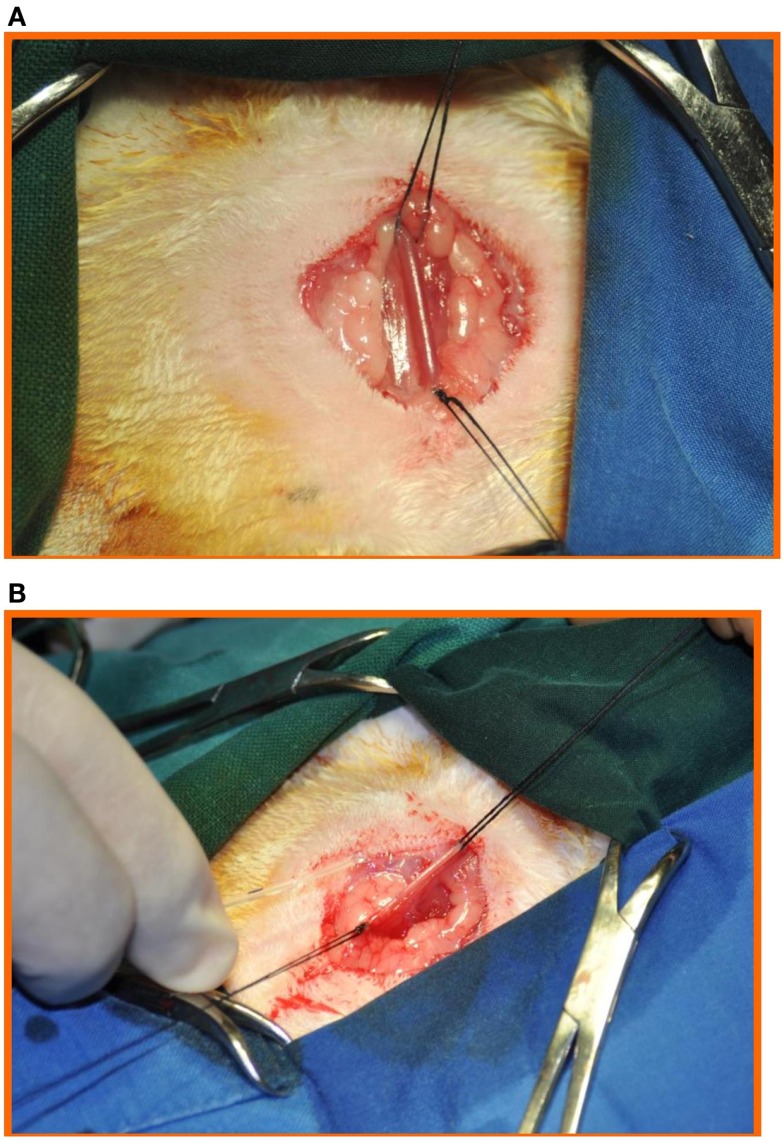
**(A)** Through a longitudinal incision in the right side of the neck, the right carotid artery was dissected free, and (**B)** distally ligated and proximally controlled with a tie.

Through a longitudinal incision in the right side of the neck, the right carotid artery was dissected free, distally ligated, and proximally controlled with a tie (Figure 2).

**Figure 3:**
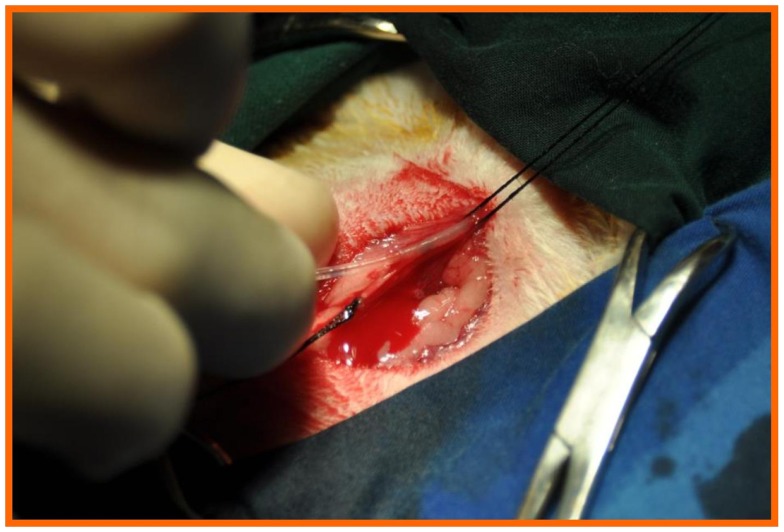
**Through a 1-mm incision on the anterior aspect of the right carotid artery, a sharp, bevel-tipped 1.22 mm catheter was introduced into the proximal right carotid artery and advanced retrograde toward the ascending aorta and connected to a pressure transducer, with a computer system for data recording**.

**Figure 4:**
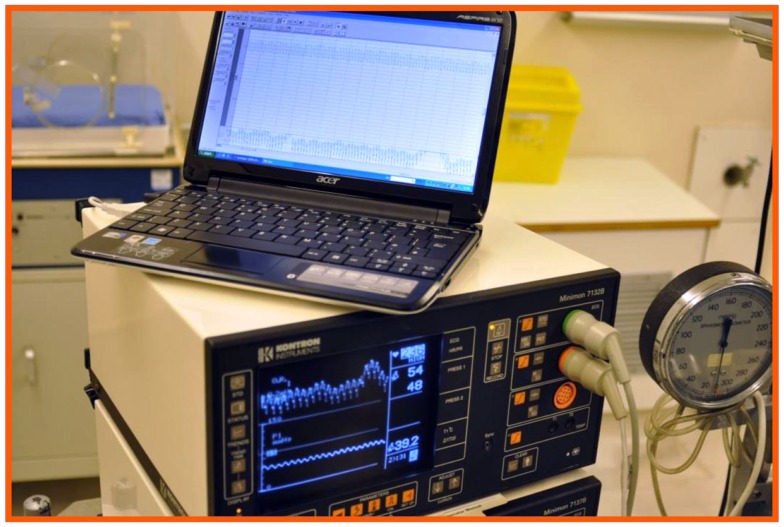
**The catheter was then connected to a pressure transducer, with a computer system for data recording**.

Through a 1-mm incision on the anterior aspect of the right carotid artery, a sharp, bevel-tipped 1.22 mm catheter was introduced into the proximal right carotid artery (Figure 3), advanced retrogradely toward the ascending aorta, and connected to a pressure transducer whose output was monitored and recorded using ACODAS (Dataq Instruments, Akron, OH, USA) (Figure 4).

### Control group

At this point for the animals in the Control group the catheter was removed from the right carotid artery, the proximal carotid artery was ligated, the tissues were closed in layers and the “sham” procedure terminated, with recovery of the animals from the general anesthesia.

### Heart failure group

In the animals in the Heart Failure group aortic valve regurgitation was obtained by disruption of ≥2 aortic leaflets by repeatedly moving the catheter across the aortic valve into the left ventricle, with continuous pressure recording. Fluoroscopy was not used. The degree of aortic valve regurgitation obtained was judged adequate when the on-line monitored pulse pressure (difference between systolic and diastolic pressure), measured through the same catheter when positioned in the ascending aorta, increased between 80 and 100% in comparison with the pre-procedure value. The maneuver was repeated until the desired degree of aortic valve regurgitation was reached.

At this point the catheter was withdrawn, the proximal carotid artery ligated, and the tissues were closed in layers. The animals were then allowed to recover from the general anesthesia.

Color Doppler echocardiography was performed in all animals of the Heart Failure group after the surgical procedure to evaluate the presence and degree of aortic valve regurgitation, LV dimensions (end-diastolic diameter), and function (shortening fraction). Aortic valve regurgitation was defined as severe in the presence of a jet >65% of the annulus.

At the end of the procedure but before recovery from anesthesia the animals were also observed for clinical signs of aortic valve regurgitation (low diastolic blood pressure, loud early diastolic murmur) and in the following 3 weeks for the appearance of clinical signs of left heart failure: respiratory distress, tachypnea, cyanosis, edema, weight loss.

### Procedure-2 = pressure overload

After an interval of 3 weeks, all animals underwent a second general anesthesia with tracheal intubation as described before.

Color Doppler echocardiography was performed in all animals before the surgical procedure.

**Figure 5:**
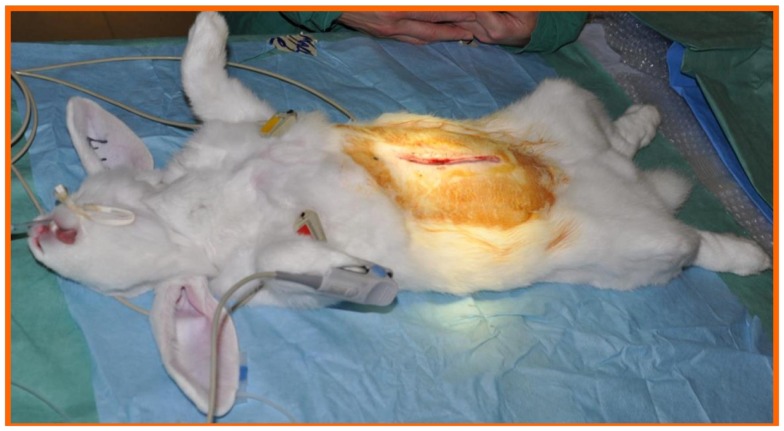
**Through a longitudinal laparotomy the abdominal aorta as well as the renal arteries were identified, and the abdominal aorta was dissected free above the renal arteries**.

Through a longitudinal laparotomy (Figure 5) the abdominal aorta and the renal arteries were identified, and the abdominal aorta was dissected free above the renal arteries.

### Control group

At this point for the animals in Control group the “sham” procedure was completed with the closure of the laparotomy incision in layers, and the animals were allowed to recover from the general anesthesia.

### Heart failure group

**Figure 6:**
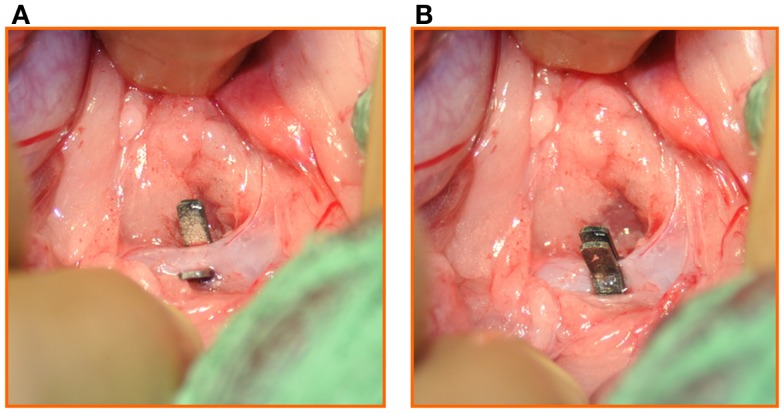
**(A)** A pre-measured (2.44 mm) silver clip was positioned around the pre-renal abdominal aorta and **(B)** occluded, inducing severe aortic narrowing with 50% reduction of the aortic diameter.

In the Heart Failure group a pre-measured (2.44 mm) silver clip was positioned around the pre-renal abdominal aorta and occluded, inducing severe aortic narrowing with approximately 50% reduction of the aortic diameter (Figure 6). At this point the surgical procedure was completed with the closure of the laparotomy incision in layers, and these animals too were allowed to recover from the general anesthesia.

Color Doppler echocardiography was performed in all animals of the Heart Failure group after the surgical procedure to evaluate the degree of stenosis created on the abdominal aorta.

At the end of the procedure the animals were observed for clinical signs of abdominal aorta coarctation (increased non-invasive diastolic pressure in the upper limbs, discrepancy between upper and lower limbs pulsatility, systolic murmur) and in the following 5 weeks for the appearance or modification of clinical signs of left heart failure: respiratory distress, tachypnea, cyanosis, edema, weight loss.

### Follow-up

In addition to the echocardiography evaluation performed before each procedure in all animals, Control and HF groups, LV size (end-diastolic diameter), and function (shortening fraction) was monitored with serial echocardiography performed after each procedure and at weekly intervals in the HF group. Five weeks following Procedure-2, after the final echocardiography evaluation, the animals were weighted, and the weight compared with the initial body weight, terminated by anesthetic overdose and the hearts were excised, weighed and sent for morphological study. Some hearts from each group were scanned by micro-CT with iodine contrast enhancement by the method previously described (38).

All procedures were carried out under the UK Home Office regulations (Animals Scientific Procedures, Act 1986).

Student’s *t* test has been used to compare the results obtained in the two groups.

## Results

Following *Procedure-1* (volume overload), while no animal in Control Group presented aortic regurgitation on evaluation by color Doppler echocardiography, all animals in the HF group developed severe aortic valve regurgitation confirmed on echocardiography by the presence of a broad jet of aortic regurgitation (nearing the aortic annulus size), with also diastolic flow reversal in the descending aorta. In the animals of the HF group an increased mean pulse pressure difference from 18 ± 3 to 38 ± 3 mmHg (*P* < 0.0001) was recorded.

After *Procedure-2* (pressure overload) in all animals in the HF group there were signs of abdominal aortic constriction: palpable thrill and audible bruit.

In the HF group one animal was terminated before the end of the protocol because of severe clinical deterioration, three animals developed pericardial effusions and three developed ascites.

### Morphology

The mean heart/body weight ratio was higher in the HF group than in the Control group (4.6 ± 0.2 vs. 2.9 ± 0.1 g/kg, *P* < 0.05), notwithstanding a significant increase in the mean body weight from the beginning of the protocol in both groups, to 3.8 kg in the HF group (*P* < 0.05) and 3.7 kg in the Control group (*P* < 0.05).

**Figure 7:**
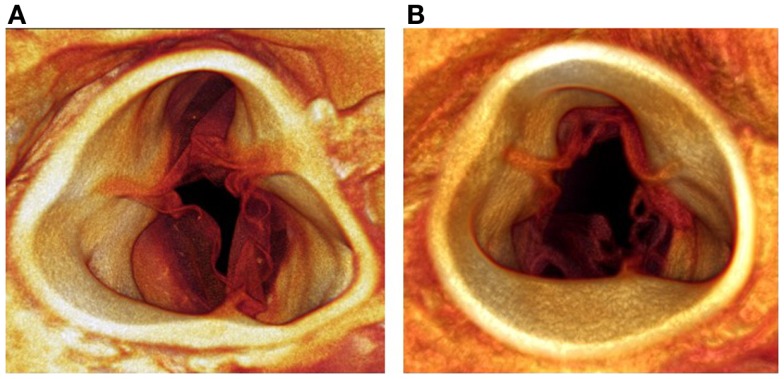
**Micro-CT images of an aortic valve from the control group (A) and from the HF group (B)**. The control valve shows leaflets with sufficient tissue for occlusion. The aortic valve from the HF group shows retraction of the leaflets toward the annulus with disruption of the leaflets.

Figure 7 shows representative micro-CT images of an aortic valve from the control group and from the HF group. The control valve shows leaflets that contain sufficient tissue for occlusion. The aortic valve from the HF group shows retraction of leaflet material toward the annulus and disruption of the leaflet structure 12 weeks after the catheter intervention.

### Function

Table 1 shows the results of the echocardiographic functional evaluation of the LV size and function performed at the end of the protocol, with significant difference (*P* < 0.0001) between the Control Group and the HF group.

**Table 1:** **Echocardiographic evaluation at the end of the protocol**.

LV end-diastolic diameter	1.49 ± 0.03 cm	2.15 ± 0.09 cm	*P* < 0.001
LV shortening fraction	41.3 ± 1.6%	26.3 ± 3.8%	*P* < 0.001

The results of the other echocardiography observations, as well as the results of histology studies on the LV morphology and the conduction tissue, will be the subject of separate reports.

## Discussion

Pressure and/or volume overload in the systemic circulation characterize the pathophysiology of several congenital and acquired cardiovascular diseases.

The consequences are LV dilatation and/or hypertrophy, with ventricular remodeling and systolic and/or diastolic dysfunction due to the structural, metabolic, and functional changes in the myocardium, as well as systemic arterial remodeling, with biochemical and humoral derangements affecting the systemic circulation.

The currently available knowledge of LV failure is derived from scattered evidence coming from experimental studies reported in the literature (1–37) as well as from the information provided by clinical observations (39–43).

There are still unanswered questions concerning the changes induced by the acute LV failure on the systolic and diastolic ventricular function, on the subsequent interaction between the two, and particularly on the effects of the time frame on the evolution and degree of the progress of the ventricular dysfunction.

This experimental model has the advantages of being reproducible, of inducing acute LV volume overload followed at fixed interval by induction of pressure overload, and is available to study the time course of LV dilatation, hypertrophy, and acute LV failure.

The documented increase of the mean heart/body weight ratio higher in the HF group than in the Control group (4.6 ± 0.2 vs. 2.9 ± 0.1 g/kg) with significant difference (*P* < 0.05) confirmed that the combined volume and pressure overloaded determined a substantial ventricular hypertrophy and dilatation.

The significant increase of the LV size and the impairment of the LV function (Table 1) proved the validity of this experimental method in creating a congestive LV failure.

In addition to the potential capability to increase the knowledge currently available on acute LV failure, our experimental model, being reproducible, allows an unlimited series of potential studies to monitor the evolution of LV failure, with the subsequent systolic and diastolic dysfunction, as well as the effects of any type of treatment, from interventional management to pharmacological administration.

Studies using this experimental model may be potentially applicable to the pediatric practice in view of the correspondence in body weight and heart size of the animals with neonates, and given similarities in the echocardiographic profile in certain types of heart failure in pediatric patients determined by severe volume and pressure overload of the left ventricle, such as the presence of an intra-cardiac left-to-right shunt associated to an obstruction in the systemic circulation (aortic coarctation with or without hypoplastic aortic arch).

Of course the realization of this experimental model requires the facilities and organization of an animal laboratory equipped with personnel and tools allowing chronic studies, and the financial and administrative support required by such this type of investigation.

## Conclusion

### This experimental model

(a) Consistently produces LV hypertrophy/dilatation and subsequent LV failure.
(b) Provides new data on time course of LV dilatation, hypertrophy, and failure.
(c) Facilitates study of the progress and evolution of LV systolic and diastolic dysfunction in the presence of induced LV failure.
(d) Is suitable to study any type of intervention or pharmacological administration to reduce the negative effects of acute LV failure.

## Conflict of Interest Statement

The authors declare that the research was conducted in the absence of any commercial or financial relationships that could be construed as a potential conflict of interest.
